# Correction: Real-world evidence of the impact of obesity on residual teeth in the Japanese population: A cross-sectional study

**DOI:** 10.1371/journal.pone.0296475

**Published:** 2023-12-22

**Authors:** The PLOS ONE Staff

## Notice of republication

This article was republished on December 13, 2023, to correct errors in the author names introduced during the typesetting process. The publisher apologizes for the errors. Please download this article again to view the correct version.
