# Supplementary figures and images for: Real-world evidence of the impact of obesity on residual teeth in the Japanese population: A cross-sectional study
Source: PLoS One. 2022 Sep 14;17(9):e0274465. doi: 10.1371/journal.pone.0274465 (PMC9473396; doi:10.1371/journal.pone.0274465)

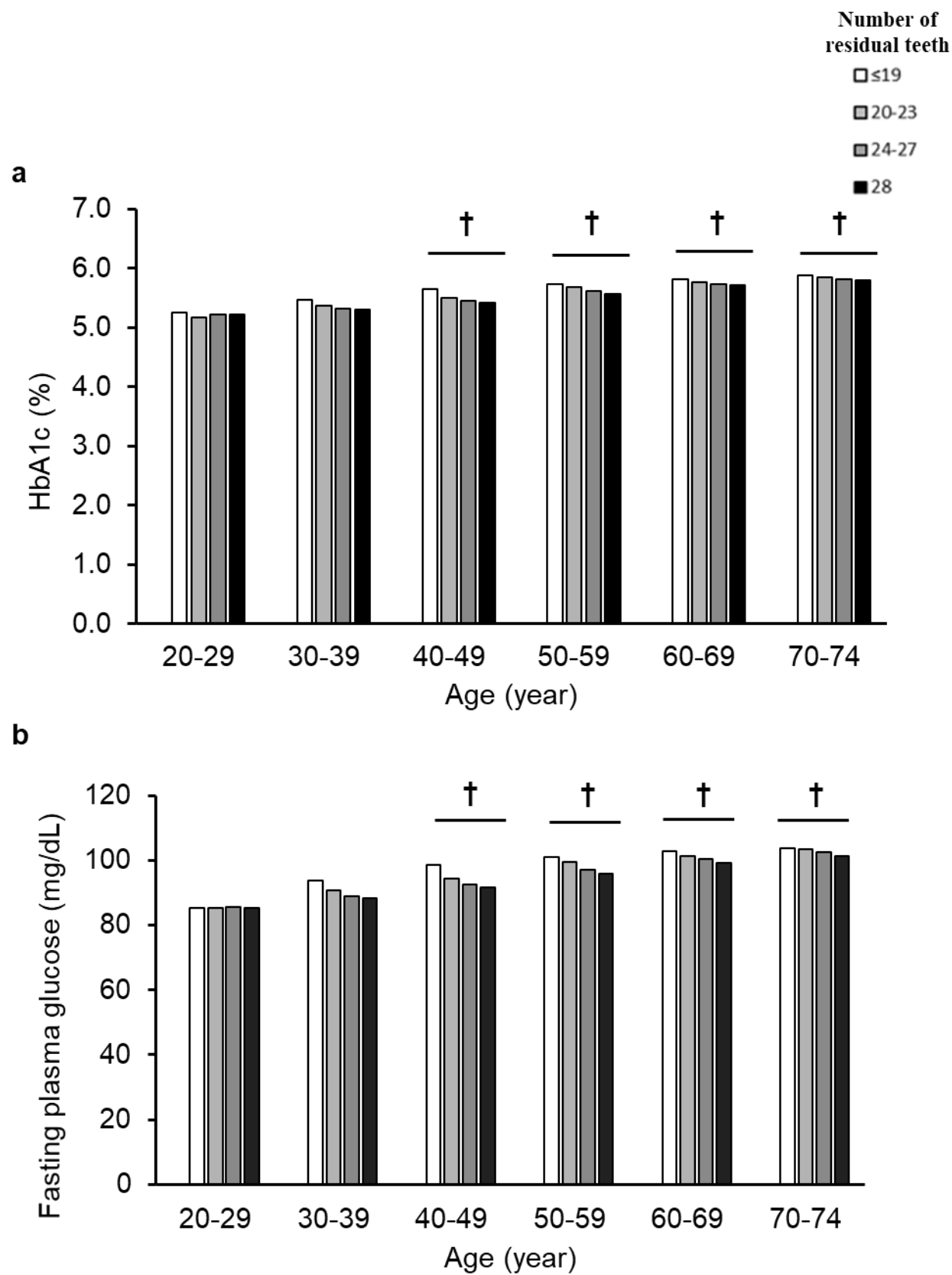

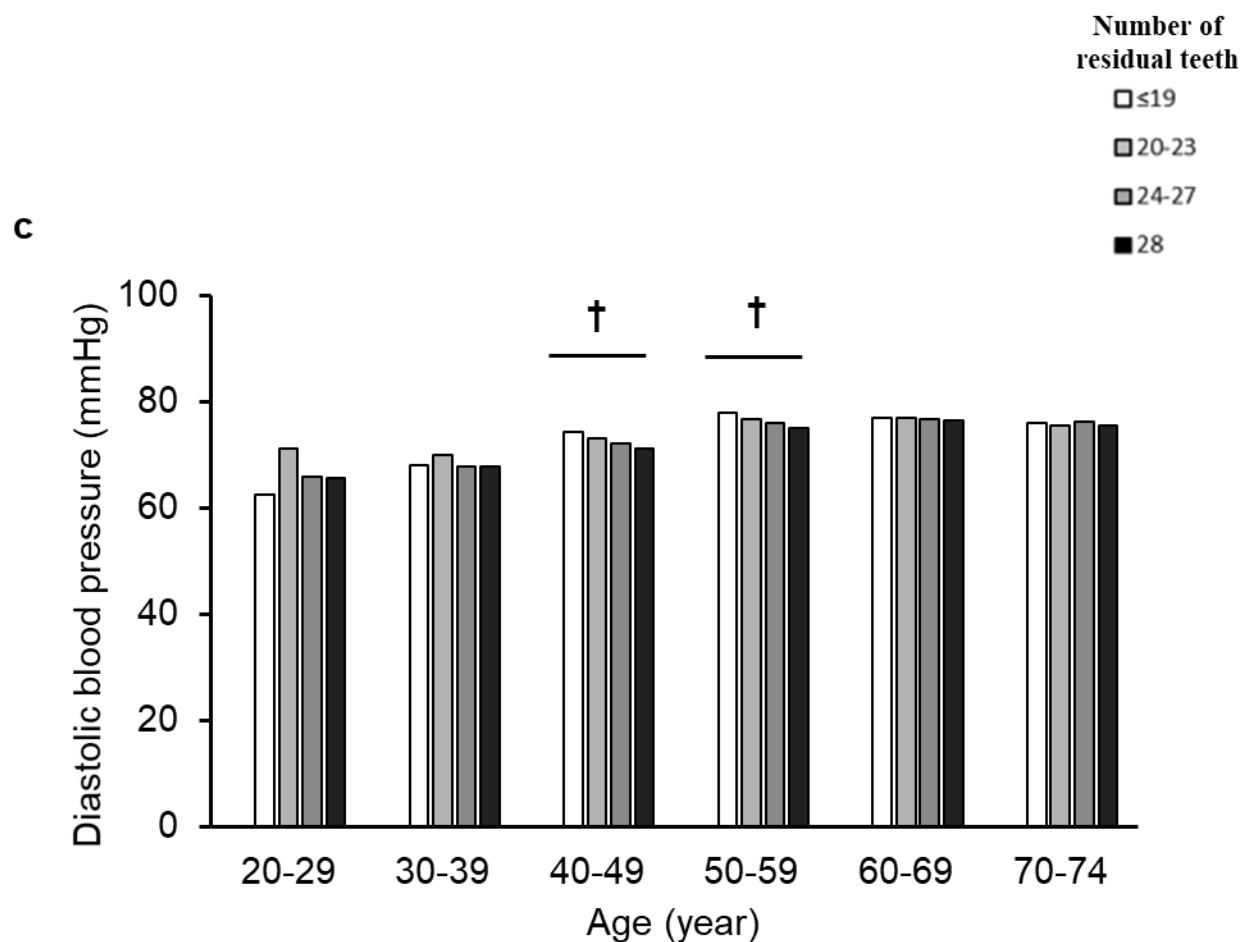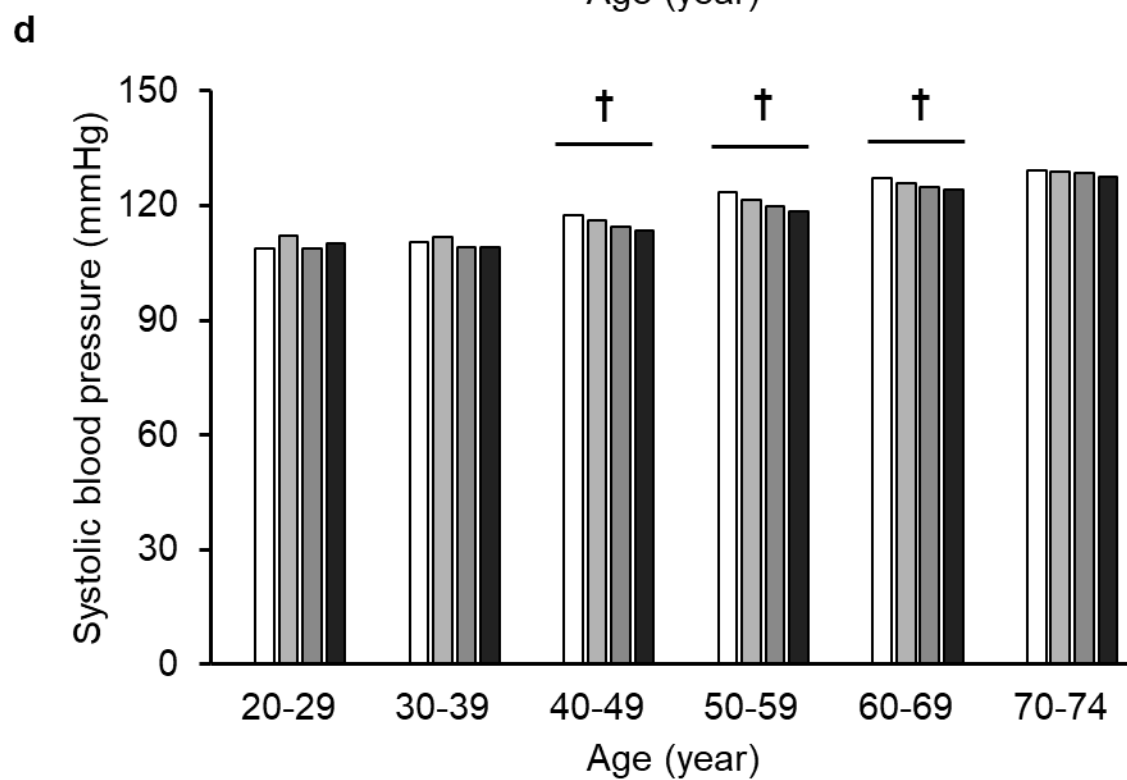

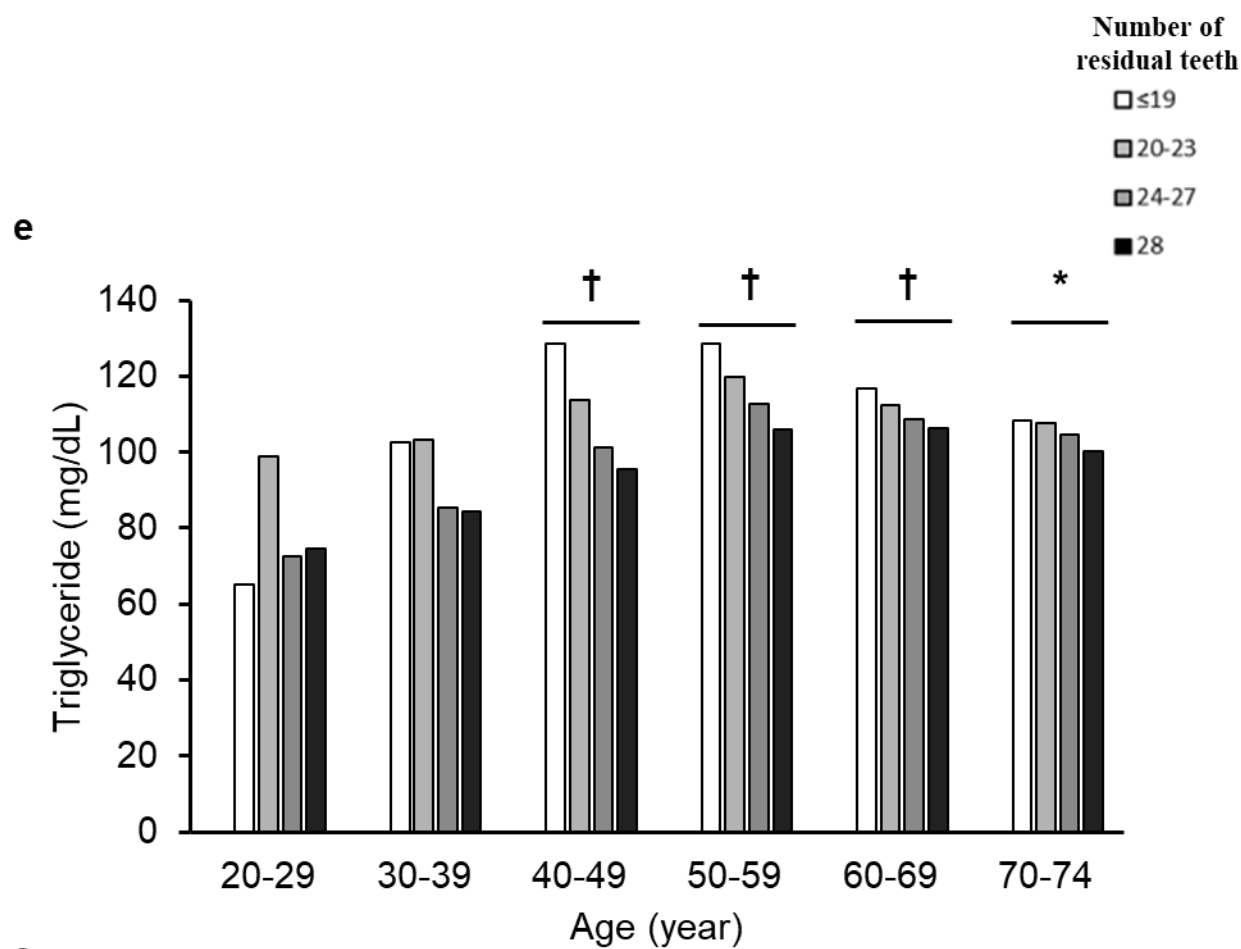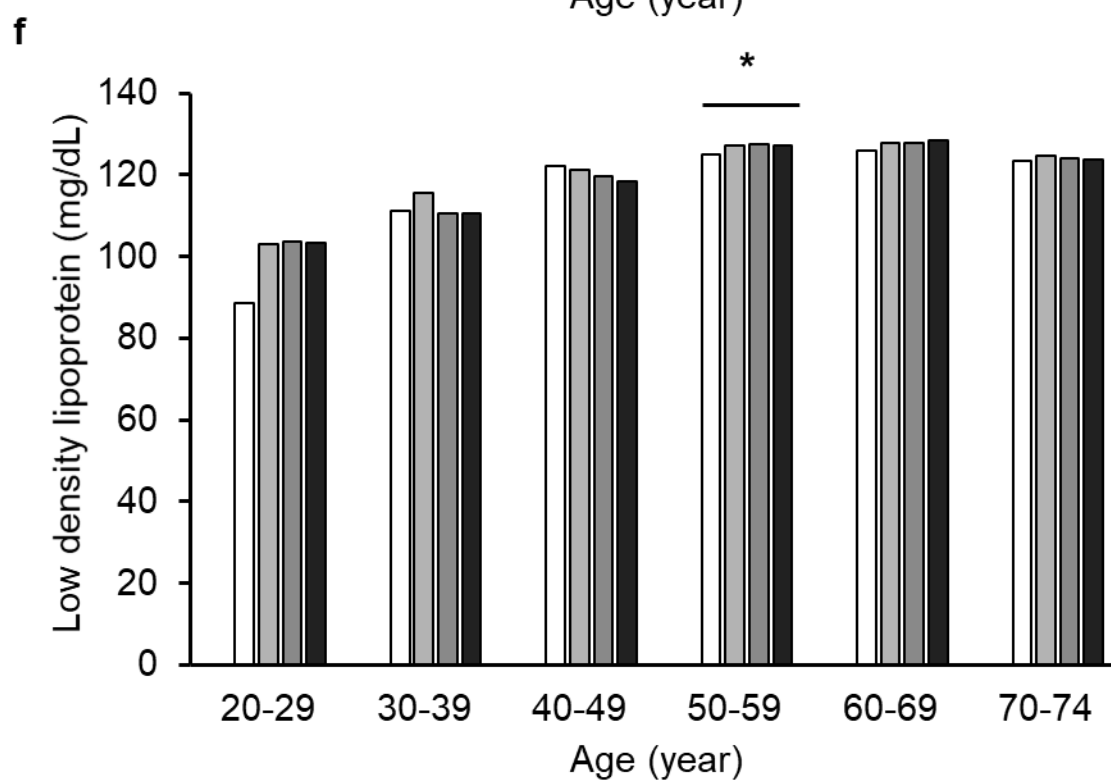

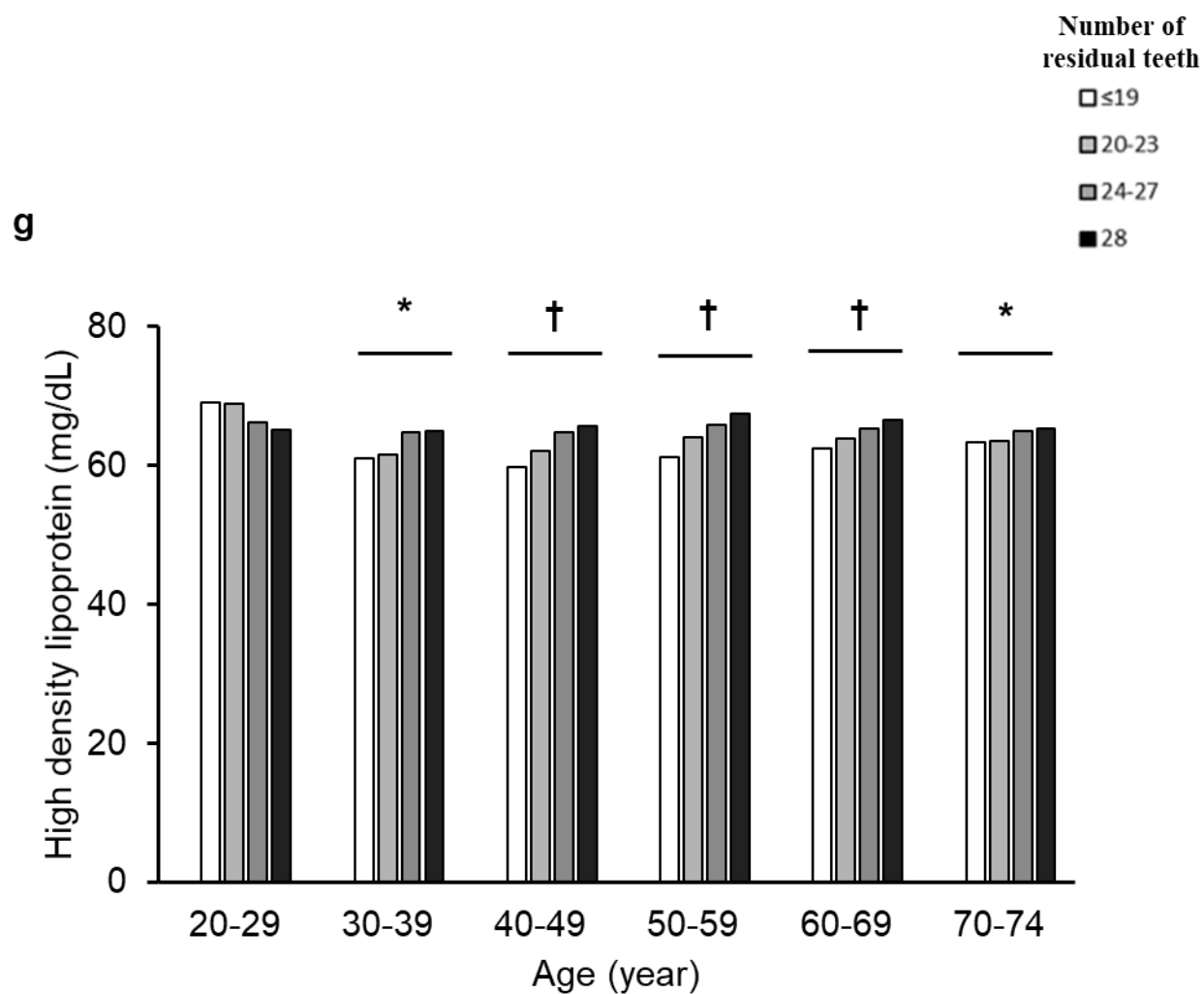

Supplement: S1 Fig — Significant linear trend across classes of residual teeth number, *; p <0.05, †; p <0.0001. (PDF) [file pone.0274465.s001.pdf]

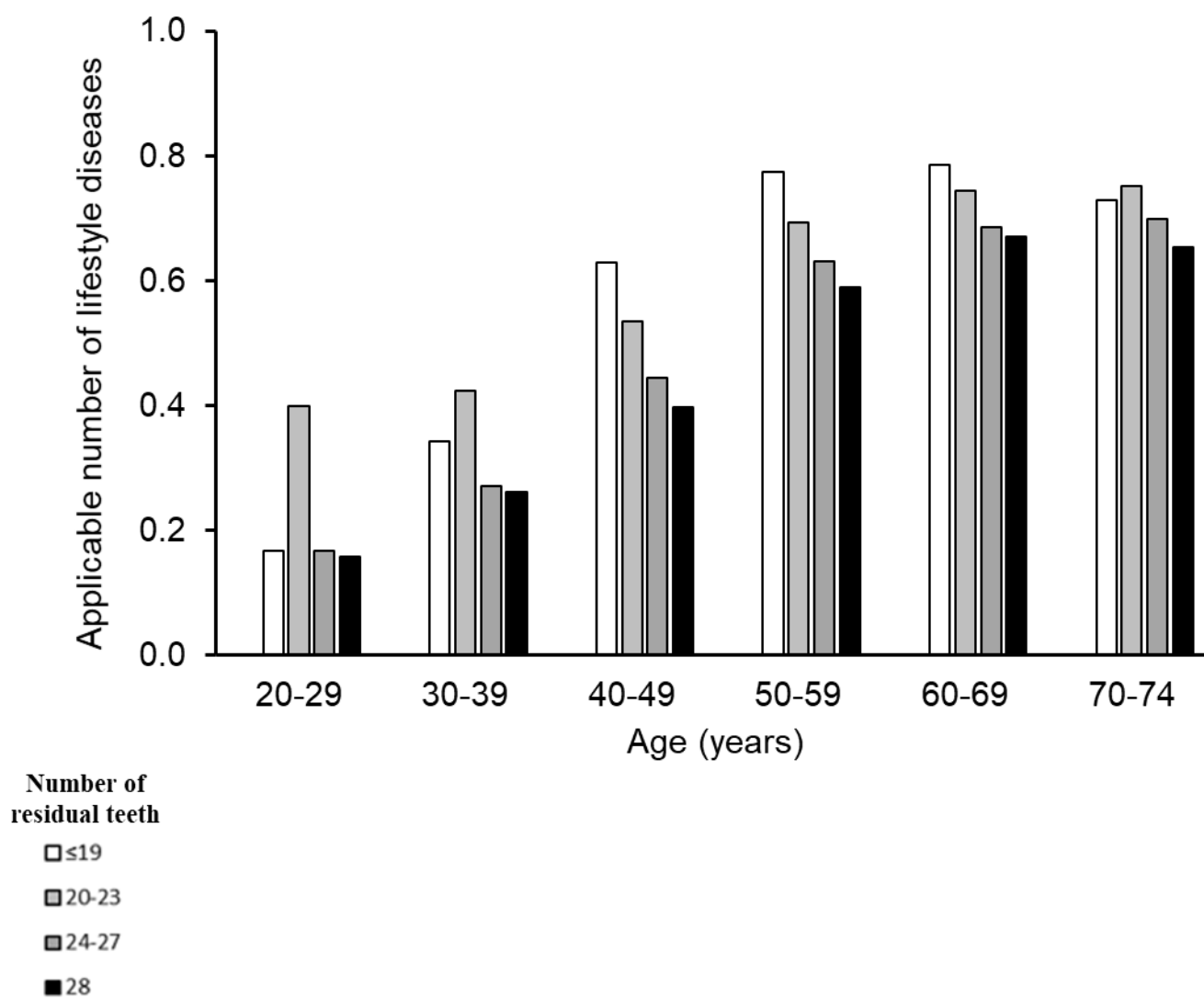

Supplement: S2 Fig — The values are the mean and sample sizes in each age—and residual teeth number- category. Lifestyle diseases refer to diabetes (FPG ≥126 mg/dL and HbA1c ≥6.5%), hypertension (SBP ≥140 mmHg and/or DBP ≥90 mmHg) and hyperlipidemia (TG ≥150 mg/dL or LDL ≥140 mg/dL or HDL <40 mg/dL). Significant linear trend across classes of residual teeth number, †; p <0.0001. (PDF) [file pone.0274465.s002.pdf]
