# Supplementary material for: Real-world evidence of the impact of obesity on residual teeth in the Japanese population: A cross-sectional study
Source: PLoS One. 2022 Sep 14;17(9):e0274465. doi: 10.1371/journal.pone.0274465 (PMC9473396; doi:10.1371/journal.pone.0274465)
Supplement: Attachment — Submitted filename: Hayashi_Response to Reviewers.docx [file pone.0274465.s004.docx]

Reviewer #1

*This manuscript if of intertest giving the ongoing debate as to the relationship between nutritional status/ anthropometrics and oral health. The study benefits from its real-world data course and large sample size.*

*Abstract: The abstract is clear and provides a useful summary of the study. However, the convulsion should be based on study findings rather than proposed future work (as this has not been evaluated).*

*Introduction: A brief succinct introduction is provided with a clear rationale for the study.*

*Methods: The study benefit from a large real-world sample of over 700,000 subjects (of which >230,000 were included in this study). In addition, a large number of variables were considered from biological to self-ratings. The statistical approach is clearly outlined and appropriate.*

*Results: A detailed results section is provided and supported by figures and tables. In additional supplementary files are available providing additional insight.*

*Discussion: A well written discussion is provided with interpretation of the findings compared with the literature. Some novel theories and aspects are provided.*

*The conclusion is adequate and this should eb used to inform the abstract’s conclusion.*

We appreciate Reviewer #1 for his/her positive and productive comments to our manuscript.

1. *Abstract: However, the convulsion should be based on study findings rather than proposed future work (as this has not been evaluated).*

We have revised conclusion section to be based on study findings as suggested (Page 2, Line 25).

We found that an increase in BMI was associated with a decrease in the number of residual teeth from younger ages independently of smoking status and diabetes in the large scale of Japanese database.

2. *Discussion: A well written discussion is provided with interpretation of the findings compared with the literature. Some novel theories and aspects are provided. The conclusion is adequate and this should eb used to inform the abstract’s conclusion.*

We have used this conclusion also in the Abstract with minor modification as suggested.

Reviewer #2:

*Few suggestions to be included*

1. *In statistical analysis, it is better to include full name of SPSS along with release date and version.*

All analyses were performed using SPSS (Statistical Package for Social Science) Statistics Ver. 26 (IBM, Inc., Armonk, NY, USA, Released on April 9, 2019), and *P*-values <0.05 were considered significant based on two-sided tests. We added these sentences in the Method section (Page 5, Line 109).

2. *According to WHO, BMI >30 is considered as obese and BMI >25 and <30 is considered as overweight. In your statistical analysis, Obesity has been considered as BMI ≥25. Please justify.*

We thank Reviewer #2 for this important point. We referred to the guidelines for the management of obesity disease 2016 (Japan Society For The Study Of Obesity) because we used a large database of the “Japanese” population. Due to the difference in the physical constitution, the guidelines indicate that regardless of gender, BMI 18.5 or more and less than 25 is defined as “normal weight” and 25.0 or more is defined as “obesity”. We added these sentences in the method section (Page 4, Line 86).
